# Twins in Dairy Herds. Is It Better to Maintain or Reduce a Pregnancy?

**DOI:** 10.3390/ani10112006

**Published:** 2020-10-31

**Authors:** Fernando López-Gatius

**Affiliations:** 1 Bovine Reproduction SLu, 22300 Barbastro, Spain; lopezgatiusf@gmail.com; 2 Agrotecnio Centre, 25198 Lleida, Spain

**Keywords:** unilateral twin pregnancies, bilateral twin pregnancies, early fetal loss, twin reduction

## Abstract

**Simple Summary:**

In dairy herds, twin or other multiple pregnancies are not desirable as they compromise the health and productive lifespan of the cows. The mean productive lifespan of primiparous and secundiparous dairy cows delivering twins is about 300 and 200 days shorter, respectively, than that of cows delivering singletons. In addition, the long-term negative effects of twinning are not limited to the early lactation but continue for a period of up to 800 days after calving. Herd management options after diagnosing a twin pregnancy are discussed from an animal health and economic perspective.

**Abstract:**

Multiple ovulations and so multiple pregnancies have increased recently in dairy cattle. The incidence of the double ovulation impact in high producers at insemination may be over 20%. Twin pregnancies are undesirable as they seriously compromise the welfare and productive lifespan of the cow and herd economy. Clinical problems extend from the time of pregnancy diagnosis to pregnancy loss, abortion or parturition. Early pregnancy loss or abortion of multiple pregnancies lead in most cases to culling. In cows reaching their term, mean productive lifespan is up to about 300 days shorter for cows delivering twins than for cows delivering singletons. While there is an urgent need to address multiple pregnancy prevention procedures in the foreseeable future, the incidence of twin pregnancies continues to rise in parallel with increased milk production. Herein, we review two contrasting measures proposed for the time of twin pregnancy diagnosis: (1) gonadotropin-releasing hormone treatment for pregnancy maintenance, or (2) embryo reduction. These options are discussed in terms of their implications for individual animal health and herd economy. Our main conclusions find that manual twin reduction has proven to be the best management option, whereas the use of prostaglandin F_2α_ for inducing abortion may be a better option than doing nothing.

## 1. Introduction

The birth of twins is a historic symbol of human fertility [1]. Even today, there are cultures that consider having twins will bring good fortune [2,3]. For the beef cattle farmer, twinning is also a very welcome event [4,5]; however, it is highly undesirable in dairy cattle. Clinical problems associated with twin pregnancies affecting dairy cows from pregnancy diagnosis to parturition have been extensively reviewed. Despite reported spontaneous embryo reduction rates of 11.2% to 28.4% occurring at around days 28–40 of gestation and usually detected at the time of pregnancy diagnosis [6,7,8], twin pregnancy losses can exceed 50%, especially during the warmer seasons [9,10]. Thus, the risk of pregnancy loss during the late embryonic/early fetal period in cows carrying twins is three to nine times greater than in cows carrying singletons. The economic burden of a dairy cow twin pregnancy in the United States has been estimated at USD 97 to USD 225 depending on the type of twin pregnancy (unilateral vs. bilateral), parity and days in milk at the time of conception; estimates of annual costs run at USD 96 million [11].

From the cow’s perspective, twinning means a high risk of being culled when pregnancy fails [11,12]. Most pregnancy losses occur within the first 60 days of gestation [13,14,15]. This is likely because this is the period in which placentation is established [16,17] and this event is also highly sensitive to any kind of stress [14,17]. Losses can be the outcome either of embryo death due to post-ovulatory oocyte ageing before fertilization, as it has been described in single ovulating cows [18], or of the inappropriate progesterone intake to two or more embryos in certain circumstances [8]. A second peak of abortions has been also described averaging at the middle of the gestation period [19]. The metabolic stress of milk production linked to a lack of space and mechanical stress may explain why the risk of abortion is greater in the middle than at the end of the lactation period. Across both these lost pregnancy peaks, unilateral twin pregnancies are the most vulnerable. In effect, losses are five to nine times higher for unilateral than bilateral twins during the late embryo/early fetal period [14,20] and abortion is 50 times more likely in cows with unilateral than bilateral twins in the middle of gestation [19]. In addition, in cows delivering twins, a very high incidence has been reported of peripartum disorders such as dystocia, stillbirth and retained placenta [21,22,23]. Each of these twinning-related disorders impairs fertility and determines a higher risk of culling after calving. For example, in an extensive study of 12,587 calving events over a period of 11 years [23], mean productive lifespan was about 300 and 200 days shorter for primiparous and secundiparous cows delivering twins, respectively, than for cows delivering singletons. Further, the long-term detrimental impacts of twinning were not limited to the early lactation but continued up to 800 days after calving.

Rather than twin pregnancy, the use of the general term multiple pregnancy would be more correct in the context of this article. However, given the numbers of triplets and quadruplets are too small to make any observable difference [24], we will only consider twin pregnancies. In fact, pregnancies with three or more embryos usually do not make it to term [25].

## 2. Management Options after Diagnosing a Twin Pregnancy

Although the decision to eliminate a cow from the herd is heavily influenced by the farmer, pregnant cows are more likely to remain in the herd. However, once a twin pregnancy has been diagnosed, the decision of what to do becomes more difficult. Veterinarians responsible for reproductive control programs should be more directly involved in decision making after diagnosing a twin pregnancy. Below, we discuss the options available. All the results and procedures described here are based on the use of ultrasonography.

### 2.1. Maintaining a Twin Pregnancy

Since inducing embryo reduction may increase the risk of pregnancy loss, cows of high genetic value or close to the culling threshold (i.e., advanced lactation) may be candidates for pursuing pregnancy maintenance. Although studies have shown that gonadotropin-releasing hormone (GnRH) or human chorionic gonadotropin (hCG) treatment given within 40 days of gestation seems not to affect the subsequent pregnancy loss rate in studies including all pregnant cows [26,27], GnRH treatment at pregnancy diagnosis promotes pregnancy maintenance, yet it has been linked to an increased rate of spontaneous twin reduction in cows carrying twins [28]. In an extensive study involving 1054 cows carrying singletons and 379 cows carrying unilateral twins, the latter were 3.2 times more likely to experience pregnancy failure than the remaining cows, whilst in cows carrying twins treated with GnRH, treatment returned similar pregnancy loss rates to those recorded in control cows carrying singletons. In this study, it was also found that GnRH-treated cows were seven times more likely to show twin reduction than control cows, with a twin reduction rate of 9.2% [28]. This effectiveness of GnRH treatment is probably attributable to the fact that either it improves luteal activity or, following the death of a co-twin, it reduces pro-inflammatory and luteolytic responses to the remains of the dead embryo, thus decreasing the pregnancy loss rate [20,28].

### 2.2. Twin Reduction

Induced embryo reduction is successful in multiple pregnancies in women [29,30,31] and mares [32,33,34]. While sophisticated techniques such as intracardiac or intrathoracic embryo/fetus puncture with or without injection of a toxic agent are used in humans, a widespread strategy among equine medicine practitioners is manual crushing of one embryo vesicle to avoid a twin pregnancy [32,33]. However, in contrast to human and equine multiple pregnancies, in twin pregnant cows, vascular embryonic fusion has occurred already before the early fetal period [25,35]. This important process of placental anastomosis is probably the reason for a high incidence of pregnancy loss after natural or induced reduction of twins in cattle. Methods proposed for twin reduction in cows on day 28–40 of gestation are manual crushing of the amniotic vesicle or transvaginal ultrasound-guided suction of allanto-amniotic fluid [20].

Before any attempt at embryo reduction, it must first be checked that both twins are alive. The presence of a dead co-twin may be detected at the time of pregnancy diagnosis and its incidence could be as high as 16% [6]. Similar results have been obtained for the methods transvaginal allanto-amniotic fluid aspiration and manual amnion rupture [10,36]. Since the aspiration-based method is ultrasound guided and requires two people, we prefer to induce embryo reduction by manual rupture of the amniotic vesicle of bilateral and some unilateral twin pregnancies within our weekly visit program in controlling reproduction. Immediately following reduction, cows receive an anti-prostaglandin treatment such as 1250 mg flunixin meglumine plus a dose of GnRH or an analogue thereof such as dephereline (i.e., 100 μg gonadorelin acetate [6-D-Phe] i.m; Gonavet Veyx, Ecuphar, Barcelona, Spain) [20]. Cumulative pregnancy loss is lower than 25% following rupture for bilateral twins and close to 40% for unilateral twins. Manual embryo reduction of twin pregnancies is today considered the most economic option to mitigate the negative impacts of twinning in dairy cows; the breakeven point for the minimum embryo reduction success rate being below 40% for multiparous and 30% for primiparous cows [11]. While there is much room for improving the results of induced embryo reduction, most current efforts target unilateral twin pregnancies.

## 3. Concluding Remarks

Twinning is considered undesirable in the dairy industry, yet from the perspective of the cow it may even be described as disastrous. In today’s world, improving the quality of life of animals has become a main focus [37,38,39]. The high incidence of dystocia, stillbirth and retained placenta should be perhaps considered a preventable consequence of management, as these disorders greatly compromise the welfare of a cow delivering twins. Manual embryo reduction has proven to be the best management option at the end of the breeding period as it diminishes the risk of late abortion and eliminates the consequences of twin births in cows that remain pregnant [11]. However, it is often difficult to balance animal welfare and our own perceptions. In some discussion forums on cow reproductive control, embryo reduction is considered both impractical and inadequate (unpublished data). For an operator with a certain amount of experience, the time between uterine horn manipulation and amnion rupture is no longer than five seconds and cows show no signs of discomfort throughout the procedure [20]. As manual embryo reduction is performed during the late embryonic period, between 28 and 40 days of pregnancy, this procedure is fully in line with European recommendations for animals used for scientific research purposes (EU Directive 2010/63/EU, 22 September 2010) and, of course, with good veterinary practices under farm conditions. In fact, as noted above, embryo reduction methods are extensively used in multiple pregnancies in women [29,30,31] and mares [32,33,34]. Last but not least, when maintaining the pregnancy is too risky and twin reduction is not feasible, the use of prostaglandin F_2α_ for inducing abortion may be a better option upon diagnosis of twins than doing nothing [11]. This is reinforced by a recent survival analysis demonstrating the enormous negative effect of transition diseases on subsequent reproductive performance [40]. Twining is not a transition disease but is the gateway for transition diseases.

During the last decades, multiple ovulations and subsequent multiple pregnancy rates have increased in parallel with milk production [20]. Although 14–15% is a common figure of dairy cows experiencing double ovulation following spontaneous estrus [41,42], the impact of double ovulation in high producers may exceed 20% [43,44,45]. This trend for the increased multiple ovulation rate over time will likely continue into the future [20]. Having discussed management options after diagnosing a twin pregnancy, the best option would still be a well-designed twin pregnancy prevention program. Promising candidate strategies for this purpose are drainage without suction of co-dominant follicles at the time of insemination or transfer of a single embryo [46,47].
